# Characterisation of PduS, the *pdu* Metabolosome Corrin Reductase, and Evidence of Substructural Organisation within the Bacterial Microcompartment

**DOI:** 10.1371/journal.pone.0014009

**Published:** 2010-11-16

**Authors:** Joshua B. Parsons, Andrew D. Lawrence, Kirsty J. McLean, Andrew W. Munro, Stephen E. J. Rigby, Martin J. Warren

**Affiliations:** 1 Centre for Molecular Processing, School of Biosciences, University of Kent, Canterbury, Kent, United Kingdom; 2 Faculty of Life Sciences, Manchester Interdisciplinary Biocentre, University of Manchester, Manchester, United Kingdom; University of California Berkeley/Joint Genome Institute, United States of America

## Abstract

PduS is a corrin reductase and is required for the reactivation of the cobalamin-dependent diol dehydratase. It is one component encoded within the large propanediol utilisation (pdu) operon, which is responsible for the catabolism of 1,2-propanediol within a self-assembled proteinaceous bacterial microcompartment. The enzyme is responsible for the reactivation of the cobalamin coenzyme required by the diol dehydratase. The gene for the cobalamin reductase from *Citrobacter freundii* (*pduS*) has been cloned to allow the protein to be overproduced recombinantly in *E. coli* with an N-terminal His-tag. Purified recombinant PduS is shown to be a flavoprotein with a non-covalently bound FMN that also contains two coupled [4Fe-4S] centres. It is an NADH-dependent flavin reductase that is able to mediate the one-electron reductions of cob(III)alamin to cob(II)alamin and cob(II)alamin to cob(I)alamin. The [4Fe-4S] centres are labile to oxygen and their presence affects the midpoint redox potential of flavin. Evidence is presented that PduS is able to bind cobalamin, which is inconsistent with the view that PduS is merely a flavin reductase. PduS is also shown to interact with one of the shell proteins of the metabolosome, PduT, which is also thought to contain an [Fe-S] cluster. PduS is shown to act as a corrin reductase and its interaction with a shell protein could allow for electron passage out of the bacterial microcompartment.

## Introduction

The two main biologically active forms of vitamin B_12_ (adenosylcobalamin and methylcobalamin) act as coenzymes and cofactors in complex rearrangement and methylation reactions, respectively [1]. The catalytic properties of these metallo-prosthetic groups arise from the cobalt-carbon bond formed with the upper axial ligand (5-deoxyadenosyl or methyl group). *E. coli* does not have the ability to synthesise adenosylcobalamin or methylcobalamin *de novo* although it does have a salvage pathway allowing the reuse of cobinamide and later intermediates [2]. Attaching the adenosyl group to the central cobalt ion of cobalamin requires reduction of the cobalt to Co(I) to generate a super-nucleophile that then facilitates the attachment of the adenosyl group from ATP with the loss of triphosphate by an adenosyltransferase enzyme [3].

Adenosylation is an important step in the biosynthesis of cobalamin [4]. An enzyme associated with the aerobic biosynthesis of cobalamin has been purified from *Pseudomonas denitrificans* with cob(II)alamin to cob(I)alamin reductase activity [5], although it is only recently that the enzyme has been identified at the genetic level in *Brucella melitensis* [6]. A detailed characterisation revealed that the protein is an NADH-dependent flavoenzyme, named CobR. This cobalamin reductase has broad substrate specificity and is able to reduce a number of corrin based substrates from Co(II) to Co(I) by a single electron reduction [6]. Technically, this reaction is challenging due to the energetically unfavourable nature of the cob(II)alamin to cob(I)alamin transition, which exhibits a reduction potential of −610 mV [7]. The enzymatic process is initiated with NADH reducing the oxidised flavin (FAD) bound to CobR. The subsequent reduced flavin promotes the single electron reduction of the central metal ion of the corrin, forming a reduced corrin and a flavin semiquinone. The remaining electron is thought to either initiate another round of reduction or to disproportionate to the flavin in the alternate active site (the enzyme exists as a homodimer) [6]. In contrast, in the anaerobic biosynthesis of cobalamin, corrin reduction is thought to be mediated by a flavodoxin [8], [9].

**Figure 1:**
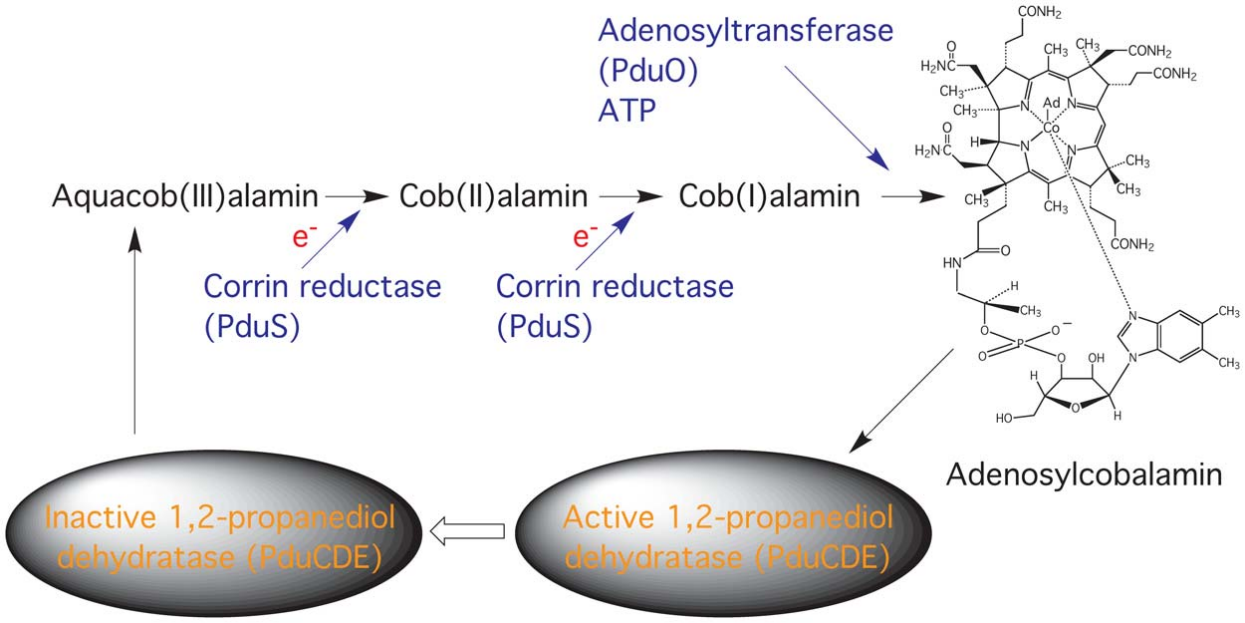
Regeneration of adenosylcobalamin from aquacobalamin. In the 1,2-propanediol utilisation metabolosome the adenosylcobalamin-dependent diol dehydratase (PduCDE) occasionally generates aquacobalamin. This inactivated form of the coenzyme is reactivated by the action of a corrin reductase, PduS, and an adenosyltransferase (PduO). This is achieved by two sequential one-electron reductions of aquacob(III)alamin to cob(I)alamin prior to the transfer of the adenosyl moiety from ATP.

PduS was identified as an enzyme encoded within the *Salmonella enterica* propanediol utilization (*pdu*) operon as another protein capable of catalysing this single electron corrin reduction [10]–[12]. It is part of a system that is responsible for the reactivation of cobalamin after it is occasionally derailed during the diol dehydratase reaction (see Figure 1) [13]. In propanediol metabolism, the enzymes responsible for the catabolism of 1,2-propanediol, including the adenosylcobalamin-dependent diol-dehydratase (PduCDE), are found encased within a proteinaceous bacterial microcompartment called a metabolosome [14]–[17]. However, the initial characterisation of the *S. enterica* PduS was restricted to work with the crude extracts containing the recombinant protein due to the low levels of protein produced [12]. More recently, the overproduction and purification of the *S. enterica* PduS has been reported [18]. Here, the purified protein was shown to contain a flavin cofactor and, in the presence of an active adenosyltransferase, was shown to be capable of mediating the generation of a cob(I)alamin species for the synthesis of adenosylcobalamin [18].

Although there is some similarity at the primary structure level between PduS and CobR, reflecting the fact that both are flavoproteins, the two are nonetheless quite distinct. CobR (18.7 kDa) is considerably smaller than PduS (48.6 kDa) and is known to be reduced by NADH. However, the N-terminal region of PduS has a possible NADH binding motif (GxGxxG) between residues 28 and 33 suggesting that it too may be a NADH flavoreductase. Bioinformatic analyses, using Expasy's ScanProsite motif finder, suggests PduS has two iron-sulphur binding motifs between residues 255–284 and 300–330 (C_264_xxC_267_xxC_270_xxxC_274_ and C_309_xxC_312_xxC_315_xxxxC_320_), regions that are absent in CobR. Thus, PduS has the potential to house up to two [Fe-S] centres. It was also suggested that the *S. enterica* PduS may also house an [Fe-S] centre [12].

In this paper we reveal that the *Citrobacter freundii* PduS [19] is an [Fe-S] containing flavoprotein that is able to catalyse both the Co(III) to Co(II) and the Co(II) to Co(I) cobalamin reductions. We also demonstrate that the protein contains two coupled [4Fe-4S] centres. Finally, we provide evidence that PduS binds to PduT, one of the shell proteins of the metabolosome [19], and we suggest that this association may represent a way to pass electrons out of the bacterial microcompartment.

## Results

### Purification of PduS

**Figure 2:**
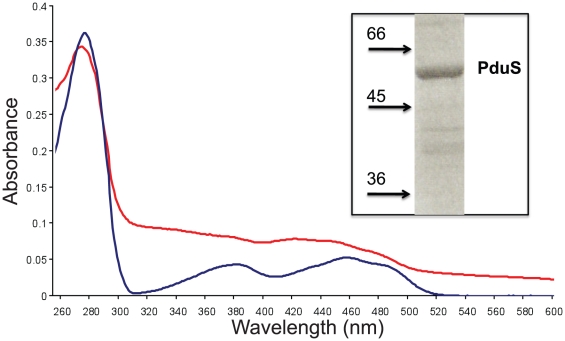
UV-visible spectrum of purified PduS. The UV-visible spectrum of aerobically-purified PduS in 20 mM Tris-HCl (pH 8.0) in red is compared to that of aerobically purified PduS (in blue). The blue spectrum is consistent with that of a flavoprotein whereas the red spectrum has added features around the 420 nm region consistent with the presence of additional [Fe-S] centre(s). A gel of purified PduS is shown in the inset.

The *C. freundii pduS* was cloned into pET14b to allow the protein to be overproduced recombinantly in *E. coli* with an N-terminal hexa-histidine tag to help facilitate purification by immobilised metal affinity chromatography (Figure 2, inset). After growth of the requisite *E. coli* strain and purification of PduS under aerobic conditions, PduS was found to be yellow in colour, indicating the presence of a flavin cofactor. In contrast, when PduS was purified anaerobically the UV-visible spectrum of the protein revealed that the flavin peaks at 380 nm and 455 nm were masked by the presence of a possible iron-sulphur cluster peak at approximately 420 nm (Figure 2) and the enzyme had a brown colour. When the anaerobically-purified protein was removed from its anoxic environment, the protein lost its brown colour and reverted to yellow. Thus, the *C. freundii* PduS would appear to be a flavoprotein with an oxygen-labile [Fe-S] centre.

The nature of the flavin prosthetic group was investigated. Purified PduS was heat-treated and the liberated flavin was analysed by reverse-phase HPLC and compared to commercially available standards of FMN and FAD. The flavin extracted from PduS was found to have a retention time identical to authentic FMN and a correspondingly similar molecular mass of M/Z 457 (M+H^+^). Thus PduS would appear to be an FMN-dependent enzyme.

### Cob(III)alamin reductase activity

To assess the PduS-dependent cobalamin reductase activity, a continuous spectrophotometric assay was employed. The reduction of the central Co(III) ion to Co(II) causes a dramatic shift in the UV-visible spectrum, where the broad absorbance band between 500 nm to 550 nm changes to a single peak at 476 nm. Purified PduS demonstrated a specific cob(III)alamin reductase activity of 701 nmol min^−1^mg^−1^ at 100 µM cob(III)alamin. Unsurprisingly, this value is far greater than the reported specific activity in a crude extract containing enhanced levels of *S. enterica* PduS [12]. The rate of reduction was calculated over a range of aquacobalamin concentrations and the enzyme displayed Michaelis-Menten kinetics. From this the enzyme was found to have a *k_cat_* of 4.04 s^−1^, and *K_M_* values for aquacobalamin and NADH of 0.59 mM and 24.7 µM, respectively.

### Cob(II)alamin reductase activity

Despite having a unique UV-visible spectrum, detecting the formation of the Co(I) nucleophile is fraught with problems due to its reactivity and short half life in solution [6]. To overcome this problem, a linked assay incorporating the adenosyltransferase (BtuR) from *Brucella melitensis* was employed. The adenosyltransferase (PduO) from the *C. freundii pdu* operon [19] was not used as the PduO was found to be insoluble. The adenosyltransferase traps the cob(I)alamin by attaching an adenosyl group, which forms the upper-axial ligand of the corrin.

Incubation of PduS with cob(II)alamin, ATP, NADH, and BtuR resulted in the formation of adenosylcobalamin, whose formation was monitored spectroscopically and verified by mass spectrometry. Adenosylcobalamin was also formed in the assay if PduS was omitted but borohydride was added in its place. The coenzyme was not formed when either PduS or BtuR were omitted from the incubation. Thus, PduS must be able to catalyse the formation of a cob(I)alamin species.

### PduS houses one or more [4Fe-4S] centres

**Figure 3:**
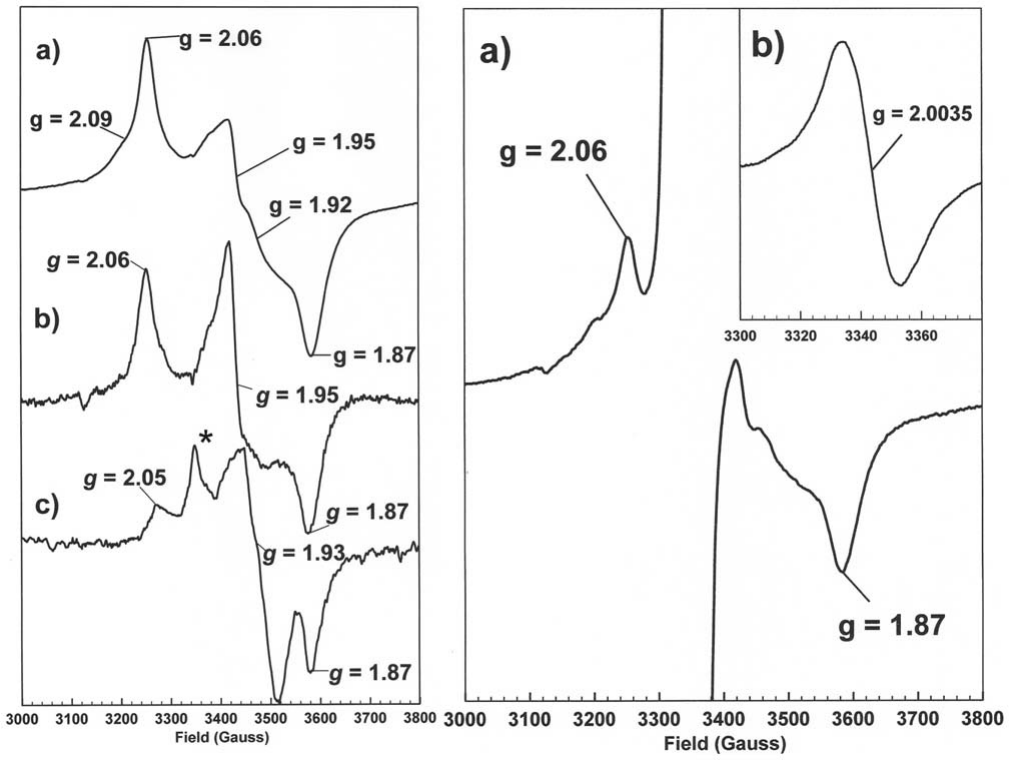
Left hand panel: X-band EPR spectra of PduS reduced using sodium dithionite, a) WT reduced using ten equivalents of dithionite showing interaction between two [Fe-S] clusters, b) WT reduced using three equivalents of dithionite, c) C270A mutant reduced using ten equivalents of dithionite (this spectrum is the sum of 16 scans), the asterisk marks a small signal from an unassigned (non-flavin) radical species. Right hand panel: X-band EPR spectra of PduS reduced using an excess of NADH, a) shows a wide sweep width with and is expanded vertically to reveal the features arising from the [4Fe-4S]^1+^ centre, b) shows a spectrum of the same sample recorded under conditions that favour the accurate registration of flavosemiquinone radical spectra and over a narrower sweep width. Experimental conditions were: microwave power 0.5 mW, modulation amplitude 5 G, modulation frequency 100 KHz, temperature 12 K for all but right hand panel spectrum b) where they were: microwave power 10 µW, modulation amplitude 1 G, modulation frequency 100 KHz, temperature 70K.

The UV-visible absorption spectrum of anaerobically prepared PduS shows a maximum at approximately 420 nm, which is typical of a protein housing a [4Fe-4S] centre (Figure 2). To confirm the presence of a suspected [Fe-S] centre and to determine the form of centre present ([2Fe-2S] or [4Fe-4S]) an EPR study was conducted. EPR samples were prepared from purified PduS (>250 µM), after reduction of the protein with dithionite (11.5 mM). Samples were frozen and stored in liquid nitrogen prior to analysis. The X-band EPR spectrum of PduS reduced by dithionite is shown in Figure 3a (left panel). This spectrum exhibits an overall rhombic lineshape with g values 2.06, 1.95 and 1.87. The spectrum was not observed at temperatures above 70K (data not shown) which, together with the g values, shows it to arise from a [4Fe-4S]^1+^ cluster, similar in EPR features to that of activated aconitase [20]. However, the spectrum actually appears to arise from two magnetically interacting (coupled) [4Fe-4S]^1+^ centres as indicated by the additional features at g values 2.09 and 1.92. It was initially thought that the magnetically coupled EPR signal was due to the protein being dimeric, but the primary sequence of PduS contains two cysteine-rich regions. Indeed, reduction of a single [4Fe-4S] cluster to the 1^+^ state was achieved by the addition of lesser amounts of dithionite giving rise to the spectrum of Figure 3b in which only the three principal g values of the rhombic [4Fe-4S]^1+^ spectrum are observed. This suggests that although the two centres are coupled, they must also possess different redox potentials indicating a more complex mechanism of electron transport within the protein than might at first be considered.

### Reduction of flavin bound to PduS

The NADH reduced X-band EPR spectrum of PduS (Figure 3 – right panel) highlights another interesting characteristic of the reductase. The spectrum shows an abundance of neutral ‘blue’ flavosemiquinone, as indicated by the isotropic EPR spectrum with a linewidth of 19.2G at g = 2.0035, suggesting incubating the protein with NADH results in net one-electron reduction of the flavin. When reduced with dithionite, no flavin signal could be observed, presumably because it had all been converted to the EPR silent hydroquinone state. During NADH reduction, some reduced [4Fe-4S]^1+^ can be observed (Figure 3). Thus it seems likely that the bound flavin is initially reduced to the hydroquinone state by 2-electron reduction by NADH. Subsequently a single electron is transferred from the flavin to the [4Fe-4S] centres resulting in formation of [4Fe-4S]^1+^ states and flavosemiquinone.

### Redox properties of PduS

It has been shown that NADH can reduce the flavin cofactor and a [4Fe-4S] cluster, but it is unknown which cofactor is the source of electrons used to reduce cob(III)alamin. To investigate this, EPR was employed since it can detect the paramagnetic form of cobalamin (cob(II)alamin) and can also monitor changes in the amount of reduced [Fe-S] and flavin semiquinone.

**Figure 4:**
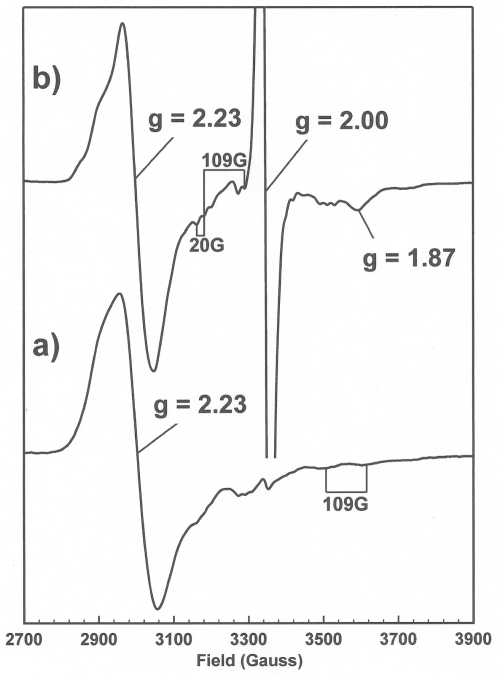
X-band EPR spectra of hydroxocobalamin and NADH in the absence, a), and presence, b), of PduS. Experimental conditions: microwave power 0.5 mW, modulation amplitude 5 G, modulation frequency 100 KHz, temperature 12K.

In the absence of PduS, a control sample containing cob(III)alamin and NADH (Figure 4) revealed that NADH has the ability to reduce cobalamin as a spectrum attributable to low spin 5-coordinate Co(II) with g**_⊥_** = 2.23, g_||_ = 2.00 and Co(II) A_||_ = 109G, signal could be seen. When PduS was added to cob(III)alamin and NADH, Figure 4, the signal changed. Cob(II)alamin could still be observed but a prominent nitrogen axial ligand to the cobalt can be seen through the resolved ^14^N 20G triplet superhyperfine splitting of the cob(II)alamin spectrum. A large amount of flavin semiquinone can also be observed as well as coupled [4Fe-4S]^1+^ signals although there is slightly less of both signals compared to the sample without cob(III)alamin. This suggests the possibility of electron transfer from the flavin or [4Fe-4S] centres to cobalamin, and/or a change in the midpoint potentials of the cofactors on cobalamin binding. The difference in the axial nitrogen ligation of cob(II)alamin in the sample containing PduS and NADH is thought to arise from the axial ligand being formed by a PduS side chain, e.g. a histidine imidazole group, as opposed to the intrinsic dimethylbenzimidazole (DMB) group. Alternatively, the geometry of the DMB may be altered in the presence of PduS leading to a modified superhyperfine coupling. The EPR spectrum in Figure 4 cannot distinguish between DMB and histidine ligation, but does show that the axial ligand of cobalamin is a nitrogen.

### Generation of PduS cysteine mutants

A comparison of PduS sequences from a number of different organisms identified nine cysteines that are fully conserved; C54, C264, C267, C270, C274, C307, C312, C315 and C320 (data not shown). With the exception of C54A, these cysteines appear to fall into two regions (C264–274 and C309–320), consistent with the protein containing more than one [4Fe-4S] cluster. Each of these cysteine residues were individually mutated to alanine, which cannot co-ordinate an [Fe-S] centre. Constructs containing these single amino acid substitutions (C54A, C264A, C267A, C270A, C274A, C307A, C312A, C315A and C320A) were generated and subsequently expressed in *E. coli*. When purified anaerobically by nickel affinity chromatography, the resulting proteins appeared colourless. This suggests the tertiary structure had changed in such a way that malformation of one [Fe-S] centre prevented the other from forming and also prevented the FMN from binding. Unfortunately this prevented absolute confirmation as to which residues co-ordinate the [4Fe-4S] cluster(s), but emphasises how crucial the native primary structure is to the activity of the enzyme. These cysteine variants did not display any cobalamin reductase activity. Analysis of these mutants using EPR spectroscopy allowed for the detection of small amounts, 3–10% of WT, of [4Fe-4S]^1+^ signal in samples of C267A, C270A, C274A and C320A reduced with an excess of dithionite. Only C270A produced sufficient [4Fe-4S]^1+^ signal for the determination of accurate g values, this spectrum is shown in Figure 3c, (left panel). This may represent the EPR spectrum of the second [4Fe-4S] cluster having the lower mid-point potential of the two such clusters as it is not seen when lower dithionite to protein ratios are used for reduction as shown in Figure 3b (left panel).

### Redox potentiometry

**Figure 5:**
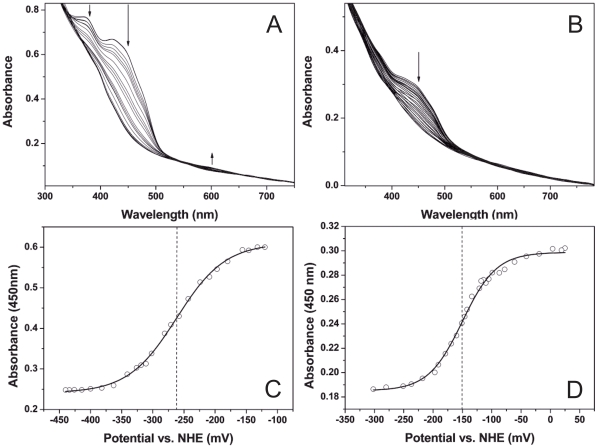
Determination of midpoint reduction potential of PduS. Spectral changes associated with the reduction of PduS (70 µM), in 100 mM phosphate buffer (pH 7.5) at 25°C with dithionite during the spectroelectrochemical titration. Spectra were recorded after the sample reached equilibrium following each addition of dithionite. The arrow indicates the wavelength at which the maximal changes in spectral properties between oxidised and reduced enzyme occur. (**a**) Shows spectra of aerobically purified PduS housing predominantly flavin during dithionite titration. (**b**) Shows anaerobically purified PduS housing [4Fe-4S] and flavin cofactors during dithionite titration. Arrows indicate directions of absorption change at selected points in these spectra. (**c**) A fit of the spectral data from panel (a) at 450 nm to the Nernst function, giving a midpoint redox potential of the cofactors to be *E^o^* = −150±5 mV. (**d**) A fit of the spectral data from panel (b) at 455 nm to the Nernst function, giving a midpoint redox potential of the flavin to be *E^o^* = −262±5 mV. Midpoint redox potentials are shown by dashed lines.

The midpoint redox potential of the flavin and the [4Fe-4S] centres of PduS were investigated by redox potentiometry. PduS was subjected to a reversible titration using dithionite as a reducing agent and ferricyanide for re-oxidation (Figure 5). The titration took approximately 4 hours, during which PduS began to aggregate slightly, which could be observed by an increase in turbidity and an increasing baseline shift. Data fits are displayed at 450 nm as the maximal changes in spectral properties between oxidised and reduced enzyme occur at this wavelength. The analysis of PduS purified aerobically, and therefore containing only the flavin cofactor, gave a value for the midpoint reduction potential of −262 mV±5 mV (Figure 5a,c). In contrast, the data collected from PduS purified anaerobically, containing both the [4Fe-4S] and flavin, gave a value for the midpoint reduction potential of −150 mV±5 mV (Figure 5b,d). The presence of the [Fe-S] centres therefore clearly influences the redox potential of the flavin.

### PduS interacts with PduT

**Figure 6:**
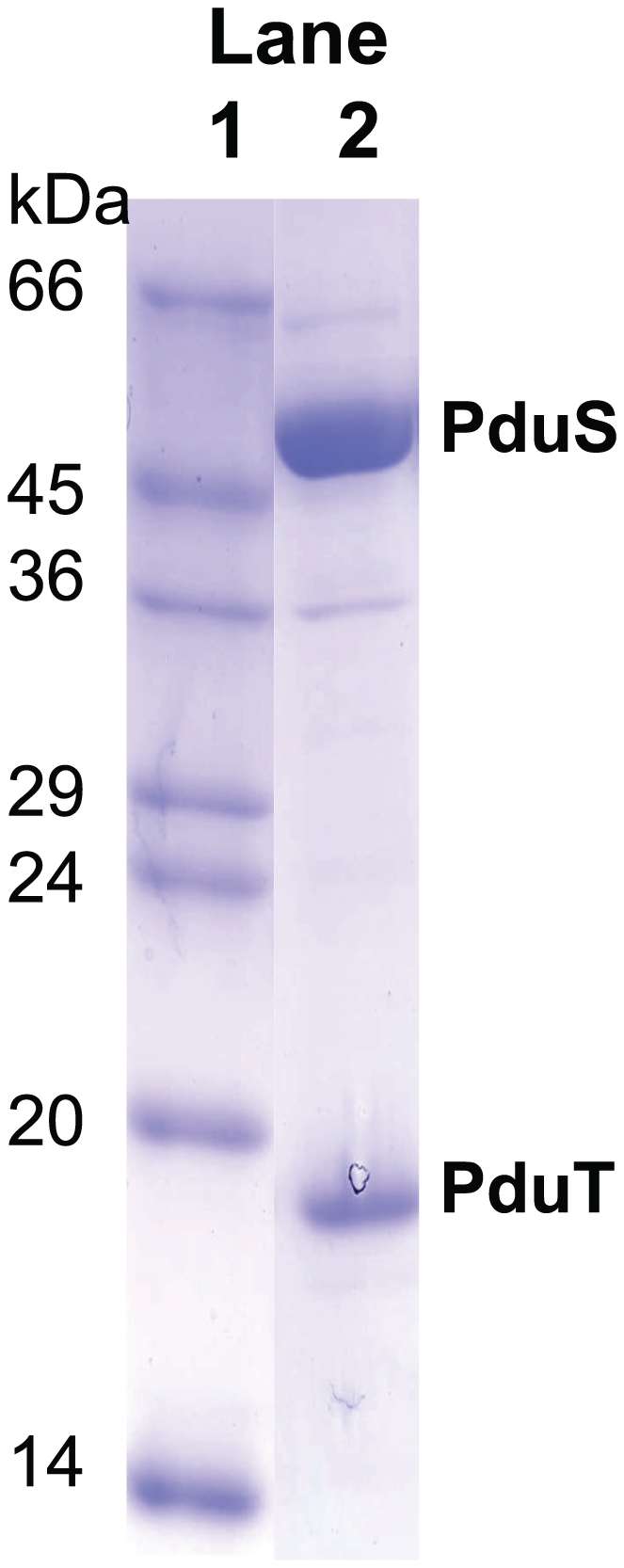
SDS-PAGE of PduS/PduT copurification. Lane 1 contains molecular mass markers as indicated. Lane 2 contains the purification eluate from a nickel column that has been applied with the crude cell extract of a strain overproducing His-tagged PduS and untagged PduT. The identities of PduS and PduT were confirmed by MALDI after trypsin digestion of the extracted protein bands.

PduT is a shell protein of the metabolosome that contains a redox cofactor, formed uniquely between four individual subunits. The [4Fe-4S] centre housed on PduT has been shown to have a redox midpoint potential of +99 mV [19]. To investigate if there is any association between PduT and PduS, the two proteins were co-produced in *E. coli* but with only PduT containing an N-terminal His-tag. Purification of the 19 kDa PduT by metal chelate chromatography resulted in the co-isolation of PduS (45 kDa), as deduced by SDS-PAGE (Figure 6). The two protein bands were excised and analysed by peptide mass fingerprinting, which confirmed their identity. In the converse experiments, when an N-terminally tagged version of PduS was co-produced with untagged PduT, no co-isolation was observed. Control experiments revealed that neither untagged PduS or PduT bind to the metal chelate column. The results indicate the PduS is able to specifically interact with PduT, and that this interaction is mediated, at least in part, via the N-terminus of PduS. Such an interaction would allow the PduS reductase to be anchored to the metabolosome shell (most likely on the interior to exchange recycled cobalamin with the propanediol dehydratase). The interaction of the corrin reductase with the shell protein PduT provides the first piece of evidence for substructural organisation inside the *pdu* microcompartment.

## Discussion

We have shown that the *C. freundi* PduS is a flavoprotein, apparently containing two coupled [4Fe-4S] centres, with the capacity to process a single electron reduction of both cob(III)alamin and cob(II)alamin. The flavin is reduced by NADH in a reaction that leads to the formation of a semiquinone, indicating that some disproportionation takes place and that electron transfer occurs to the [Fe-S] clusters.

It has been suggested that PduS is not actually a corrin reductase, but is in reality a flavin reductase to produce free dihydroflavin that then mediates the corrin reduction when the corrin is bound to the adenosyltransferase [18]. The thinking behind this suggestion is compelling since, when bound to the adenosyltransferase, the cob(II)alamin is held in a 4-coordinate manner, which has the desired effect of raising the cob(II)alamin to cob(I)alamin couple by around 250 mV to −360 mV [21]–[23]. This change in potential then allows the reduction to be facilitated by free reduced flavin and as the substrate is held on the transferase it also ensures that the nucleophilic cob(I)alamin species is rapidly quenched by adenosylation. In this respect it is argued that PduS is just an electron transfer protein rather than a bespoke corrin reductase [18].

However, in this study we have shown by EPR that cobalamin is able to bind to PduS. Such an interaction is more consistent with PduS being a specific corrin reductase since it involves molecular recognition of cobalamin. In reality, therefore, cobalamin reduction may involve a complex assembly with interactions between PduS and the metabolosome adenosyltransferase (PduO) allowing for enhanced corrin reduction activity. Indeed, a very recent publication has shown that PduS and PduO do, in fact, interact and that PduS is part of a cobalamin recycling system within the metabolosome [24]. We have not observed PduS under turnover conditions, and therefore do not know if there is a 4-coordinate cob(II)alamin species formed under such conditions. Furthermore, we have not observed such a species under our steady state conditions with limiting reductant, but such a species need not be formed in high yield in order for the reduction process to proceed. Is a reductase that binds a cob(II)alamin substrate an electron transfer protein or a cob(II)alamin reductase? We would suggest the latter, the alternative is that PduS functions as an electron transfer protein that happens to bind to and provide an axial ligand to cob(II)alamin. We find the argument for a cob(II)alamin reductase persuasive, but the details of the mechanism are yet to be revealed.

Within the metabolosome PduS has an important role to play in the reactivation of the corrin coenzyme, which can become deadenosylated during the catalytic cycle of the diol dehydratase to form a cob(III)alamin species [13]. The reactivation requires two one-electron reduction steps to permit the adenosylation and regeneration of the coenzyme form (Figure 1). This has to happen in the microcompartment and indeed both PduS and PduO are located within the metabolosome [24]. The role of PduS may therefore be to provide the dihydroflavin to allow these reductions [18] but also to provide a way of channelling unwanted electrons to the outside of the compartment, employing its [Fe-S] centres to absorb free electrons not utilised in corrin reduction and to pass them onto PduT. As part of the outer shell of the supermolecular metabolosome complex PduT may be able to transfer electrons onto other acceptors found in the cell cytoplasm.

## Methods

### Chemicals

Most chemicals were purchased from Sigma. Other materials were provided by the following suppliers: restriction enzymes, modification enzymes, were from Promega and New England Biolabs UK; chelating-Sepharose fast flow resin was from GE Healthcare, Little Chalfont, Bucks, UK; pET3a, pET14b and pLysS were from Novagen, Madison, WI; tryptone and yeast extract were from Oxoid, Basingstoke, UK; sodium dithionite was from Roche, Poole, UK; and oligonucleotide primers were from Invitrogen, Paisley, UK.

### Strains and cloning

To enable high level expression in *Escherichia coli*, *pduS* was amplified by PCR using pAR3114 as a template and 5′-CGCCATATGAAGACAGCTATGACGGCAG-3′ as the forward primer and 3′CGCACTAGTTAACCTCTTATTACCGTGATGGATTGC 5′ as the reverse primer. The PCR fragment was digested with the appropriate restriction enzymes and ligated into pET14b to generate pJP024.

### Recombinant Protein overproduction and purification

The pJP024 plasmid containing the *pduS* gene was transformed into *E. coli* BL21(DE3)pLysS and grown in LB containing 100 mg/litre ampicillin and 33 mg/litre chloramphenicol with aeration at 37°C. As the culture reached an OD_600_ of approximately 1, protein production was induced by the addition of 400 µM isopropyl 1-thio-β-D-galactopyranoside (IPTG). The cultures were subsequently left to grow overnight at 16°C. The cells were harvested by centrifugation (4,000×*g*, 15 minutes) and resuspended in 10 ml binding buffer (20 mM Tris-HCl pH 8.0, 500 mM NaCl, 5 mM imidazole). Cells were lysed by sonication and immediately centrifuged to remove insoluble debris (36,000×*g*, 15 minutes). To preserve any possible [Fe-S] centres the resulting lysate was transferred to an anaerobic glove box (Belle Technology) containing less than 3 ppm oxygen. The soluble lysate was applied to an immobilised nickel affinity column and washed with increasing concentrations of imidazole until the protein was eluted from the column in 1 M imidazole. The *B. melitensis* BtuR (adenosyltransferase) was purified as previously described [6].

### Identification of flavin bound to PduS by HPLC

The bound flavin cofactor was extracted from purified PduS by heating the protein at 100°C for 10 minutes. Subsequently, the precipitate was removed by centrifugation leaving a yellow supernatant. The flavin was then detected by an HPLC coupled to a micrOTOF-Q (Bruker) mass spectrometer, equipped with online diode array and fluorescence detectors, as described previously [6]. The retention time, spectral characteristics and mass spectrum of the cofactor extracted were compared to authentic standards of FMN and FAD.

### PduS cob(III)alamin reductase assays

The reduction of cob(III)alamin to cob(II)alamin was measured using a spectrophotometric assay. Assay mixtures contained 20 mM Tris-HCl pH 8.0, 0.5 mM NADH and cob(III)alamin in a total volume of 1 ml. Assays were carried out under anaerobic conditions inside a nitrogen purged glove box (Belle Technology). Assay components were dispensed into a UV capable cuvette, placed in a Hewlett Packard 8452A photodiode array spectrophotometer and assays performed in duplicate. The assay mixture was placed in the peltier of the photodiode array for 5 min to allow the temperature to reach 37°C. Cob(III)alamin reduction was initiated by the addition of PduS. Initial rates were determined by monitoring the decrease in absorbance at 525 nM and using Δε_525_ = 4.9 mM^−1^cm^−1^ for calculations. The initial rates of reduction were calculated over a range of cob(III)alamin concentrations and plots of initial rate against substrate concentration were generated to show typical Michaelis-Menten kinetics. Kinetic parameters were determined by non-linear regression.

### PduS cob(II)alamin reduction

A linked assay that incorporates the adenosyltransferase (BtuR) was employed to confirm cob(II)alamin to cob(I)alamin reductase activity of PduS. This system uses an ATP-dependent adenosyltransferase to convert any cob(I)alamin formed into the respective adenosyl derivative. Assay mixtures containing combinations of 20 mM Tris-HCl pH 8.0, 100 µM cob(II)alamin, 0.4 mM ATP, 1 mM MgCl_2_, 0.5 mM NADH, 250 µg of BtuR and 500 µg of PduS were incubated in an anaerobic glove box and left in the dark overnight to prevent photolysis prior to RP-HPLC-MS analysis. Controls with borohydride in place of PduS (positive) and without either PduS or BtuR (negative) were also performed.

### Electron Paramagnetic Resonance Spectroscopy (EPR)

Samples were prepared as described in the text and then frozen in liquid nitrogen. EPR experiments were performed on a Bruker ELEXSYS E500 spectrometer operating at X-band employing a Super High Q cylindrical cavity (Q factor ∼16,000) equipped with an Oxford Instruments ESR900 liquid helium cryostat linked to an ITC503 temperature controller.

### Redox potentiometry

Redox potentiometry was performed essentially as described previously [25]. Redox titrations were performed in a glove box under a nitrogen atmosphere, and all solutions were degassed under vacuum with argon. Oxygen levels were maintained at less than 2 ppm. The protein in 100 mM phosphate buffer (pH 7.0) with 10% glycerol was titrated electrochemically according to the method of Dutton [26] using sodium dithionite as the reductant and potassium ferricyanide as the oxidant. Mediators (2 µM phenazine methosulphate, 5 µM 2-hydroxy-1,4-naphthoquinone, 0.5 µM methyl viologen and 1 µM benzyl viologen) were included to mediate the range between +100 to −480 mV, as described previously [25]. At least 15 minutes were allowed to elapse between each addition of reductant to allow stabilisation of the electrode. Spectra (300–800 nm) were recorded using a Cary UV-50 Bio UV-visible spectrophotometer via a fibre optic absorption probe immersed in the enzyme solution. The electrochemical potential of the solution was measured using a Hanna pH 211 meter coupled to a Pt/Calomel electrode (ThermoRussell Ltd.) at 25°C. The electrode was calibrated using the Fe^3+/2+^ EDTA couple (+108 mV). A factor of +244 mV was used to correct relative to the standard hydrogen electrode.

### Site directed mutagenesis of PduS

The cysteine residues thought to be responsible for co-ordinating the iron-sulphur centre were mutated by site directed mutagenesis. Each targeted cysteine was mutated to an alanine residue, which cannot co-ordinate an [Fe-S] centre. Constructs showing single amino acid substitutions (C54A, C264A, C267A, C270A, C274A, C309A, C312A, C315A and C320A) were generated by use of the Quikchange II site directed mutagenesis kit (Stratagene). The mutants were prepared using pJP024 as a template and primers listed in Table S1. The sequences were confirmed as correct by sequencing the inserts in both directions using standard T7 promoter and T7 terminator primers.

### PduT - PduS interaction study

An interaction between PduS and PduT was investigated by looking for copurification of the two proteins from an *E. coli* strain producing both proteins. This was achieved by transforming *E. coli* with pJP028, which harbours *pduT* with sequence for an N-terminal His-tag encoded on pLysS, and pJP077, which encodes an untagged version of *pduS* on pET3a. The transformants were grown in 1L of Luria-Bertani medium until an OD_600_ of 1.0 when protein production was induced with 400 µM IPTG at 16°C overnight. The cells were harvested by centrifugation (4,000×g at 4°C for 10 minutes) and resuspended in 10 ml binding buffer (20 mM Tris-HCl pH 8.0, 0.5 M NaCl, 10 mM imidazole). The cells were lysed by sonication (six 30 seconds bursts, with 30 second cooling intervals on ice at an amplitude of 65%) and insoluble debris removed by centrifugation at 37, 000×g for 15 minutes. The supernatant was applied to a chelating sepharose column charged with Ni^2+^. Once bound to the column, the crude extract was subjected to washes containing stepwise increases in imidazole concentration up to 400 mM. All wash fractions were analysed by SDS-PAGE to confirm co-elution of the His-tagged and untagged protein. The identities of the eluted bands were confirmed by peptide mass fingerprinting as previously described.

## Supporting Information

Table S1 (0.13 MB DOCX)
