# Supplementary material for: Characterisation of PduS, the pdu Metabolosome Corrin Reductase, and Evidence of Substructural Organisation within the Bacterial Microcompartment
Source: PLoS One. 2010 Nov 16;5(11):e14009. doi: 10.1371/journal.pone.0014009 (PMC2982820; doi:10.1371/journal.pone.0014009)
Supplement: Table S1 — (0.13 MB DOCX) [file pone.0014009.s001.docx]

Supplemental Information for

**Characterisation of PduS, the *pdu* metabolosome corrin reductase, and evidence of substructural organisation of the enzyme within the bacterial microcompartment.**

by

Joshua B. Parsons, Andrew D. Lawrence, Kirsty J. McLean, Andrew W. Munro, Stephen E. J. Rigby, Martin J. Warren

Table S1. List of primers used in the generation of the following mutants of PduS: C54A, C264A, C267A, C270A, C274A, C309A, C312A, C315A and C320A. The mutations were generated by use of the Quikchange II site directed mutagenesis kit (Stratagene). The mutants were prepared using pJP024 as a template and primers below.

| CBFpduS_C54A_forward | 5’CAATGCGGCAGAAGCTGAGCCGATGCTG 3’ | TGT changes to GCT |
| --- | --- | --- |
| CBF*pduS_C54A_reverse* | 3’CAGCATCGGCTCAGCTTCTGCCGCATTG 5’ | ACA changes to AGC |
| CBF*pduS_C264A_forward* | 5’CGCAAAAACCGTTGCCGAGCAGTGTCGG3’ | TGC changes to GCC |
| CBF*pduS_C264A_reverse* | 3’CCGACACTGCTCGGCAACGGTTTTTGCG5’ | GCA changes to GGC |
| CBF*pduS_C267A_forward* | 5’CCGTTTGCGAGCAGGCTCGGTTGTGTACGG 3’ | TGT changes to GCT |
| CBF*pduS_C267A_reverse* | 3’CCGTACACAACCGAGCCTGCTCGCAAACGG 5’ | ACA changes to AGC |
| CBF*pduS_C270A_forward* | 5’GCAGTGTCGGTTGGCTACGGATTTGTGCC 3’ | TGT changes to GCT |
| CBF*pduS_C270A_reverse* | 3’GGCACAAATCCGTAGCCAACCGACACTGC 5’ | ACA changes to AGC |
| CBF*pduS_C274A_forward* | 5’GTGTACGGATTTGGCCCCAAGACATTTG 3’ | TGC changes to GCC |
| CBF*pduS_C274A_reverse* | 3’CAAATGTCTTGGGGCCAAATCCGTACAC 5’ | GCA changes to GGC |
| CBF*pduS_C309A_forward* | 5’CGGCCCTGACCGCTTCAGAATGCAATG3’ | TGT changes to GCT |
| CBF*pduS_C309A_reverse* | 3’CATTGCATTCTGAAGCGGTCAGGGCCG5’ | ACA changes to AGC |
| CBF*pduS_C312A_forward* | 5’CCTGTTCAGAAGCCAATGTATGTG3’ | TGC changes to GCC |
| CBF*pduS_C312A_reverse* | 3’CACATACATTGGCTTCTGAACAGG5’ | GCA changes to GGC |
| CBF*pduS_C315A_forward* | 5’GAATGCAATGTAGCTGAAAGCGTAGC3’ | TGT changes to GCT |
| CBF*pduS_C315A_reverse* | 3’GCTACGCTTTCAGCTACATTGCATTC5’ | ACA changes to AGC |
| CBF*pduS_C320A_forward* | 5’GTGAAAGCGTAGCCGCTCCGGTTGGGATTTC 3’ | TGT changes to GCC |
| CBF*pduS_C320A_reverse* | 3’GAAATCCCAACCGGAGCGGCTACGCTTTCAC 5’ | ACA changes to GGC |
